# Toward a Better “Person–Environment Fit” through Items Calibration of the SIS-C

**DOI:** 10.3390/ijerph17103471

**Published:** 2020-05-15

**Authors:** Víctor B. Arias, Antonio M. Amor, Miguel A. Verdugo, María Fernández, Benito Arias, Alba Aza

**Affiliations:** 1 Institute on Community Integration, Department of Personality, Assessment and Psychological Treatments, Faculty of Psychology, University of Salamanca, 37005 Salamanca, Spain; vbarias@usal.es (V.B.A.); verdugo@usal.es (M.A.V.); mariafernandez@usal.es (M.F.); azhernandez@usal.es (A.A.); 2 Institute on Community Integration, Department of Psychology, Faculty of Education and Social Work, University of Valladolid, 47011 Valladolid, Spain; barias@psi.uva.es

**Keywords:** context-based intervention, social-ecological model of disability, person–environment fit, supports paradigm, support needs, support needs assessment, quality of life, rights, Supports intensity scale (SIS)

## Abstract

The Supports Intensity Scale–Children’s Version (SIS-C) is the only available tool to assess extraordinary support needs for children and adolescents with intellectual disability. In past years, several works have proclaimed the need for its ongoing improvement as a measurement instrument. To contribute to this line of research, the goal of this work is to analyze the reliability of the SIS-C and its usefulness to distinguish between different levels of intensity of support needs. To address this, 814 children and adolescents with intellectual disability (M = 11.13 years; SD = 3.41) were assessed using the SIS-C Spanish version. Item response theory analyses were conducted to estimate latent scores and assess measurement quality along the support needs continuum. The SIS-C items showed good overall discrimination and information values, and none showed problems that required their removal or modification. However, all the scales composing the SIS-C showed problems in discerning high levels of intensity of support needs, especially for children and adolescents with severe/profound intellectual disability. This ceiling effect may be an obstacle for both research and practice involving the SIS-C. Implications for research and practice are discussed, and future lines of research for improving the SIS-C are provided.

## 1. Introduction

Framed in a social-ecological approach and in a strengths-based perspective, the supports paradigm conceives intellectual disability (ID) as a state of functioning characterized by a mismatch between the persons with ID competencies and the environmental demands, defined by the contexts of participation and the age and culturally appropriate activities to develop in such contexts [1]. This mismatch originates support needs, understood as a “psychological construct referring to the pattern and intensity of supports necessary for a person to participate in activities linked with normative human functioning” [2] (p. 135).

Leaving behind the concept of ID as a deficit within the person and emphasizing the interaction person–environment is the defining characteristic of the supports paradigm [1]. From this paradigm, it is assumed that all persons have support needs because everyone experiences mismatches in certain situations or activities. Thus, through the lens of the supports paradigm, the main difference between persons with and without ID concerns the nature of their support needs. In this sense, persons with ID, given that they experience intense and ongoing mismatches, have extraordinary support needs that extend beyond what most typically functioning people need to participate in the same contexts and activities [2].

Beyond this conceptualization, the supports paradigm has also brought a renewal in professional practices in the field of ID. Contrary to approaches focused on the functional rehabilitation of persons with ID [3], the supports paradigm emphasizes the “support needs assessment and planning” process. Through this process, persons with ID define first, without restrictions, those contexts and activities in which they would like to participate following their vital expectations. These contexts and activities are the bases to determine the pattern and intensity of the extraordinary support needs that persons with ID require in order to offer them personalized supports. Supports are defined as “resources and strategies that aim to promote the development, education, interests, and personal well-being of a person and that enhance individual functioning” [2] (pp.135). Supports can be classified according to their nature and focus. Thompson et al. [2] distinguish between personal supports (i.e., those including natural or informal supports and paid or professional supports), technological supports (i.e., including high technology, like a wheelchair; medium technology, like an app; or low technology, like the use of reminders), and environmental adaptations, modifications and accommodations. According to their focus, supports can be person-directed (i.e., those whose focus is to build upon the strengths of the person) or be directed at modifying environmental demands and tasks, making them more accessible [1]. No matter their nature and focus, from this social-ecological perspective, supports are bridges that maximize the person-environment fit, and aim to meet the needs and improve the functioning and participation of persons with ID in their life project–which is critical for them to achieve personal desired outcomes that increase their quality of life (QoL) [1,3,4,5]. In a nutshell, the supports paradigm allows the building of bridges for persons with ID to be causal agents over their lives, consistent with the United Nation’s Convention on the Rights of Persons with Disabilities [6].

The importance of the support needs construct within the supports paradigm has motivated a growing emphasis on its measurement. Although there exist different approaches to assess support needs, the current trend focuses on developing standardized measures of extraordinary support needs for persons with ID based on the supports paradigm [7,8], such as the Service Needs Assessment Profile [9]; the Instrument to Classify Support Needs for People with Disability [10]; and the Supports Intensity Scale [11], updated as the Supports Intensity Scale–Adult’s Version (SIS-A) [12]. Of these, the SIS-A is the most used at the international level, translated into 13 languages and used in 16 countries according to the American Association on Intellectual and Developmental Disabilities (AAIDD) [13]. The SIS-A allows the measurement of the extraordinary support needs that adults with ID aged 16–64 require to participate in 49 activities in six contexts of daily living: Home living, Community living, Lifelong learning, Employment, Health and safety, and Social activities. To assess extraordinary support needs, each activity is rated following three measurement methods: frequency, daily support time, and type of support. Several works have reported evidence on the good psychometric properties of the scores obtained using the SIS-A (for an in-detail study, see [7]).

The proliferation of standardized measures of extraordinary support needs, such as the SIS-A [12], has facilitated the implementation of the supports paradigm. These tools have not only boosted the support needs assessment and planning process [8], but also improved the efficiency of resources allocation at organizational level, a critical resource to optimize supports planning for improving the QoL of persons with ID [14,15]. Given that support needs assessment and planning should be started as soon as possible and be offered through the lifespan of the person with ID [16], the AAIDD, given the lack of standardized measures of extraordinary support needs for children and adolescents with ID and taking as a reference the good psychometric properties reported in relation to the SIS-A, developed and validated the Supports Intensity Scale–Children’s Version (SIS-C) [17], the version for children and adolescents of the SIS-A.

The SIS-C (described subsequently in the instrument section) was designed to assess extraordinary support needs in children and adolescents with ID between 5 and 16 years [17]. Starting from the hypothesis of the influence of age on support needs (i.e., the younger the children, the stronger their support needs), the standardization sample for developing norms was stratified following six age cohorts (i.e., 5–6, 7–8, 9–10, 11–12, 13–14, and 15–16). The standardized portion of the tool aims for the assessment of the extraordinary support needs that children and adolescents with ID may require to participate in 61 activities in seven contexts: Home living activities (HLA), Community and neighborhood activities (CNA), School participation activities (SPA), School learning activities (SLA), Health and safety activities (HSA), Social activities (SA), and Advocacy activities (AA). As in the SIS-A [12], each activity is rated across three measurement methods (i.e., type of support, frequency, and daily support time) and the scores obtained through the use of the SIS-C count with evidence of validity and reliability [7]. The SIS-C has been adapted and validated in different contexts (e.g., [18]) and there is an increasing interest in investigating its appropriateness and evidence of validity and reliability for children and adolescents with other disabilities [19,20,21].

Since its availability, the SIS-C [17] has been used by various studies for support needs assessment and planning for children and adolescents with ID. For example, Walker et al. [22], on the basis of the SIS-C use, developed personalized educational plans that, rather than focusing only on student’s literacy, aimed to enhance other relevant personal outcomes such as participation in their community. Recently, Schalock, van Loon et al. [23] used the SIS-C to inform a personalized support plan for improving the QoL of a student with ID in inclusive settings in the Netherlands. However, despite these works, use of the SIS-C is still not as generalized as that of the SIS-A [24], so efforts are being made to promote its use within general education settings to enhance outcomes for students with ID [25].

Notwithstanding the evidence on the use of the SIS-C and the good psychometric properties reported for its use with children and adolescents with ID [7], a critical issue concerning the SIS-C use is its refinement as a measurement instrument—a need that has already started to be addressed by research. Specifically, one line of research that has gathered much attention is the analysis of the SIS-C properties by considering the three measurement methods used to score support needs [26,27]. Seo et al. [26] explored seven multitrait–multimethod (MTMM) models, each one including a substantive trait (i.e., the support needs domain) and three method factors matching the three measurement models used in the assessment (i.e., type of support, frequency, and daily support time). The authors found that, in general, the factorial loadings of the substantive traits were significantly higher than those of the method factors (*p* < 0.01), except for the items of the CNA and AA domains, whose average factorial loadings were significantly higher for the “daily support time” method than for the substantive trait. With these results, the authors concluded that the MTMM model used demonstrated enough evidence of convergence validity to support the use of the raw scores obtained through the three measurement models used in the SIS-C. 

Although the previous study highlighted the existence of effects associated with the measurement methods, it did not yield enough information to make decisions based on the relevance of such effects. A way to facilitate the interpretation of the MTMM model used in the previous research would be through the quantification of the size of the factors in terms of explained variance and the proportion of reliable variance. With the evidence of the abovementioned study, the interpretation of the bifactor model needed the quantification of its impact on the unidimensionality of the model and of the reliability with which the substantive trait reproduces the target construct. Starting from this rationale, Verdugo et al. [27] replicated the previous work in Spain and extended it by analyzing the relevance of the method effects to determine whether these effects hindered a precise support needs assessment, and if they were relevant enough to change the way individual scores are produced or even to generate modifications to the SIS-C. To address this, the authors used a bifactor model as an approximation to analyze the MTMM and monotrait–heteromethod matrices. Essentially, the findings were similar to those reported by Seo et al. [26] regarding the location of the main sources of method variances, with the CNA and HSA domains the most affected and the method variance concentrated in the daily support time items. The implications of this work are relevant, indicating that the raw scores obtained through the daily support time measurement method are significantly contaminated by the variance unrelated to the support needs construct [27].

The cited studies have focused on investigating the construct validity of the SIS-C through the comparison of different factorial structures. One aspect that has been less studied is the reliability of the SIS-C and its utility for the purposes for which it was designed. Traditionally, the extent to which a set of items works well is judged by a single index such as the alpha coefficient [28]. Here, the standard error of measurement (SEM) of the assessed person is inversely related to the reliability coefficient so that the reliability and the standard error are constant for all the persons, no matter their score in the test of their real level in the construct. However, it is more realistic and valid to assume that a measure will present different accuracy levels at different ranges of the latent variable [29,30,31,32,33] so that a measurement instrument may be reliable for a certain range of the latent variable but not very reliable in others, as is usually the case, for example, in clinical screening instruments [34].

That said, the main goal of the SIS-C is to assess extraordinary support needs to provide personalized systems of supports to enhance the person’s QoL [8]. Consequently, it is expected that the SIS-C assesses with reliability a wide range of intensity of support needs so that most children and adolescents with ID are evaluated with enough reliability no matter the intensity of their support needs. However, to the best of our knowledge, there are no works that have investigated the reliability of the SIS-C at different ranges of support needs. Is the SIS-C reliable enough for the assessment of support needs at all ranges of support needs? How accurately does the SIS-C capture individual differences in the continuum of support needs? Is the SIS-C useful in the case of all the children and adolescents with ID, or is it only useful for certain ranges of the intensity of support needs? Is it necessary to reformulate, remove, or add items to improve the functioning of the SIS-C? Answering these questions will generate a better knowledge of the psychometric properties and utility of the SIS-C, as well as suggest improvement actions for its refinement—all being critical issues for improving current practices regarding support needs assessment and planning for children and adolescents with ID. With the purpose of addressing these questions, this study aimed to analyze the SIS-C reliability and its usefulness in distinguishing between different levels of intensity of support needs. Specifically, we used models based on item response theory (IRT) to obtain the location and discrimination parameters of the 61 activities (i.e., items) of the SIS-C. IRT does not conceptualize the reliability as a constant property for all the assessed persons, but rather allows the measurement error to vary along the latent continuum depending on the interaction between the properties of the items and the characteristics of the persons. This, along with the possibility of estimating the amount of information provided by each item, will allow us to obtain relevant insights regarding the quality and the usefulness of the measure for its purposes.

## 2. Materials and Methods 

### 2.1. Instrument

The SIS-C is the only standardized measure available to assess extraordinary support needs in children and adolescents with ID aged 5–16 years [17]. As mentioned (see Introduction), the tool is normed following age bands (i.e., 5–6, 7–8, 9–10, 11–12, 13–14, and 15–16). Regarding its structure, the SIS-C comprises two parts. The first part consists of eight items from which to make an a priori estimation of the probable extraordinary support needs that the child or adolescent with ID may have in seven daily living contexts and in general. The second part has two sections: (a) a section related to “Exceptional Medical and Behavioral Support Needs” and (b) the “Support Needs Scale.” The Exceptional Medical and Behavioral Support Needs section identifies specific medical conditions (e.g., parenteral feeding) and challenging behaviors (e.g., truancy) that may require substantial levels of support, regardless of the relative intensity of support needs required by the person. These conditions are rated following a three-point Likert rating scale, where higher scores indicate greater needs (i.e., 0 = No support needed; 1 = Some support needed; 2 = Extensive support needed).

The Support Needs Scale is the standardized section of the SIS-C. It includes seven daily living contexts and is designed to assess extraordinary support needs that children and adolescents with ID require to participate in 61 activities related to such contexts. Table 1 describes each activity domain along with the three measurement methods used to rate the intensity of the extraordinary support needs for these domains.

Each activity is scored by adding the value given to each measurement method (i.e., each activity can have a score between 0 and 12). Raw scores are transformed into standard scores for each domain. The sum of the standard scores allows calculation of the “Support Needs Index,” a measure of the intensity of the extraordinary support required by the child or adolescent with ID. As the SIS-C is normed following age bands, the standard scores and the Support Needs Index give information on the percentile of each child or adolescent, making it possible to know where he/she is along the support needs continuum regarding his/her same-age peers with ID for each domain and globally. The standard scores for each domain also allow calculation of the “Support Needs Profile,” which gives information on the distribution of the extraordinary support needs across the domains [17].

The SIS-C follows a semi-structured interview implemented by a qualified interviewer to at least two informants [17]. This semi-structured interview involves an interaction between the interviewer and the informants, and its adequate functioning is necessary to gather an accurate estimation of the extraordinary support needs that the child or adolescent with ID may have. There are different considerations for this semi-structured interview to be regarded as adequate [8,17]: (a) the interviewer’s knowledge on the supports paradigm and in the SIS-C; (b) the establishment of a good rapport between the stakeholders; (c) the interviewer’s ability to guide the conversations elicited during the interview; and (d) the characteristics and composition of the informants. First, the interviewer should be familiar with the supports paradigm, with the SIS-C goal, and he/she should have completed at least a four-year degree. Second, it is common that the informants, especially when they meet for the first time with the interviewer, feel anxious or that they have the wrong expectations about the interview or the meaning of the SIS-C assessment. Paying attention to the respondents’ concerns and doubts, showing flexibility, and adjusting their expectations about the SIS-C goals are chief to establish a good rapport. Third, the interview should flow as a conversation, it being the role of the interviewer to help the respondents focus on the SIS-C purpose whenever necessary. It is the responsibility of the interviewer to gather the relevant information on the extraordinary support needs that the child or adolescent with ID may have for all the SIS-C activities and domains. To gather this information, the interviewer asks the informants about the “extra” support that the child or adolescent with an ID of a certain age band (e.g., 14–15 years) requires beyond the support that a typically developing same-age peer would need to participate in the activity. The interviewer also has to clarify the different measurement methods and the rating scale options and, if there is a disparity between the informants regarding the support needs of the assessed person, then it is necessary to discuss and reach an agreement between all those present in the interview. Finally, regarding the composition and the nature of the informants, it is better to select a dyad of informants composed by a professional and a family member to have a more complete picture of the extraordinary supports required by the child or adolescent with ID in all the SIS-C domains. To be selected as informants, they must have known the assessed child or adolescent for at least three months, and they must have had recent interactions with him/her in the contexts included in the SIS-C [35].

The standardized section of the SIS-C, specifically, its translation and validation in Spanish [18], was used to address the aim of this paper. The SIS-C Spanish follows Tassé and Craig’s guidelines to adapt standardized measures to different contexts from the original [36] and the recommendations of the International Test Commission [37]. As in the original version, the SIS-C Spanish has optimal evidence of validity and reliability regarding its scores [7].

### 2.2. Participants

The use of the SIS-C to assess extraordinary support needs involves, beyond interviewers, the assessed children or adolescents with ID and the informants reporting on their support needs [17].

A total of 814 children and adolescents with ID were assessed (M = 11.13 years; SD = 3.41). The students who were assessed comprised a heterogeneous sample according to sociodemographic and clinical variables, as detailed in Table 2.

Regarding the informants, most were professionals, essentially teachers (63%), psychologists (8%), speech therapists (6%), therapeutic pedagogy teachers (5%), therapists (3%), physiotherapists (2%), Snoezelen therapists (1.6%), and caregivers (1.4%). On average, these informants had known the children or adolescents with ID for more than 3 months (M = 3.31 months). Following the SIS-C guidelines [17], we sought to have at least two informants for each assessment. This was possible for 732 interviews. For these interviews, the additional informants were family members for 460 cases (mothers in 84% of interviews).

### 2.3. Procedure

Participants were recruited following a non-probabilistic convenience sampling method. To recruit participants, the research team wrote a letter presenting the research project and the inclusion criteria to participate (i.e., as interviewer, informant, or student with ID). The letter was sent by e-mail to various organizations, schools, and high schools providing supports to children and adolescents with ID (who were between 5 and 16 years) in the 17 Spanish Autonomous Communities. The research team received a positive answer from 48 organizations from 10 Autonomous Communities, each one providing, on average, 15 participants (ranging from 3 to 63). Subsequently, the organizations willing to participate were contacted by telephone, and informed consent forms and all the details regarding the project were sent to them to share with the families and/or legal guardians of the children and adolescents to be assessed.

Once the consent forms were filled, the assessments of children and adolescents with ID started. Approximately one-third of the assessments were conducted by the research team. When it was not possible, the research team asked the organizations to select one person who would serve as the interviewer within the organization. Then, a twofold measure was taken to ensure consistency between the interviews conducted by the research team and those done by the interviewers selected by the organizations. First, the research team requested the organizations to select as the interviewer a person who matched the requirements stated in the SIS-C for the interview process (see Instrument section) [17]. On the other hand, the selected interviewers were trained by a research team member through face-to-face meetings or via online training sessions. Training consisted of training sessions on the supports paradigm and how to implement the SIS-C. The training sessions included both theoretical content and role-playing exercises. Once the assessments were completed, all data were introduced in a database and were treated for their subsequent analyses.

All the procedures described in this paper followed the ethical standards required by research that involves human participants. As a requisite to start the research, the research project was assessed and approved by the Bioethics Committee of the University of Salamanca (resolution available upon request to the contact author). This research also followed the standards on data protection in force in Spain, aligned with the General Regulation on Data Protection of the European Union (Regulation EU2016/679), so alphanumeric codes were assigned to all the participants to guarantee their anonymity. All procedures comply with the principles of the 1964 Declaration of Helsinki and its amendments.

### 2.4. Data analysis

All data were analyzed with the software IRTPRO 4.0 using the graded response model (GRM) [38]. The GRM assumes, in addition to the usual IRT assumptions, that the categories in which the child or adolescent is rated can be ordered, as is the case, for example, with the probabilistic rating scales of summative estimates or “Likert type.” The model aims to obtain more information than if the response levels were only two (e.g., yes or no) and, in this sense, it is an extension of the two-parameter logistic model (2-PLM) to ordered polytomous categories.

The GRM specifies a person’s probability of being rated with an *ik* category or higher as opposed to being rated with a lower category when the rating scale has at least three categories, and is expressed as:

(1) $$P_{ik}^{*}(\theta_{j})=\frac{e^{D_{\alpha i}(\theta_{j}-\beta_{ik})}}{1+e^{D_{\alpha i}(\theta_{j}-\beta_{ik})}}$$

(2) $$P_{ik}^{}(\theta_{j})=P_{ik}^{*}(\theta_{j})-P_{ik+1}^{*}(\theta_{j}),$$

where *k* is the ordered response option, $P_{ik}^{}(\theta_{j})$ is the probability of answering to option *k* in item *i* with a latent trait level $\theta_{j}$, $P_{ik}^{*}(\theta_{j})$ is the probability of answering to option *k* or higher in item *i* with a latent trait level $\theta_{j}$, $\theta_{j}$ is the person’s latent trait level, $\beta_{ik}$ is the location parameter of alternative *k* of Item *i*, $\alpha_{i}$ is the discrimination parameter of Item *i*, and *D* is the constant 1.702.

Prior to estimating the GRMs, it was verified that the data met the criteria of unidimensionality and local independence through optimized parallel analysis [39], the estimation of the explained variance by the first factor in exploratory factor analysis (i.e., unweighted least squares), and the indices of closeness to unidimensionality based on minimum rank factor analysis recommended by Ferrando and Lorenzo–Seva [40]: the explained common variance (ECV; values above 0.85 suggests that data can be treated essentially as unidimensional) and the mean of item residual absolute loadings (MIREAL; values below 0.30 indicate the lack of relevant sources of local dependency). Moreover, for each SIS-C domain, the strength and the replicability of the unidimensional model and the quality of the individual scores were assessed through the generalized replicability of the latent construct (H-G) [41], the factor determinacy index (FDI) [42], and the marginal reliability calculated from the estimated a posteriori scores. H-G values above 0.80 indicate that the factor is robust and stable across samples and studies. FDI values higher than 0.90 suggest that the factor scores are good proxies of the latent scores in the factor. Values of marginal reliability above .80 mean that the scores have been estimated with enough accuracy.

## 3. Results

### 3.1. Unidimensionality, Local Independency, and Quality of the Measure

Table 3 shows the results of the previous analyses for the estimations of the GRMs. For all the SIS-C scales (hereafter, the term “domain” is substituted by “scale,” a term more accurate for this field of data analysis), the parallel analysis recommended retaining a single factor. The first eigenvalue of the unidimensional models retained between the 79% (SPA and SA) and the 87% (HLA) of the total variance of data. For all cases, the ECV values were above 0.90 (M = 0.94) and the MIREAL values were below 0.20 (M = 0.16). The FDI and H-G indices and the marginal reliability were above 0.95 for all cases. All these results suggest that all the scales acquired high unidimensionality, that there was no evidence on strong violations of local independency, and that data presented high quality and reliability.

As these results were considered adequate to guarantee the unidimensionality and quality of the data, we then proceeded to estimate the GRMs.

### 3.2. Estimation of Graded Response Models

Table 4 shows the estimated parameters for each scale. The alpha parameter represents the capacity of the item to discern between different levels of the latent variable (i.e., support needs). The items obtained alpha values between 2.21 (“sleeping and/or napping” in HLA) and 5.96 (“taking action and attaining goals” in AA), with a mean of 4.02. Given that α_i_ values of 0.01–0.24 can be considered very low, values of 0.25–0.64 low, values of 0.65–1.34 moderate, values of 1.35–1.69 high, and values ≥ 1.7 very high [43], it can be concluded that, in absolute terms, the discrimination parameters were very high for all the items of the seven scales. Likewise, the standard errors were low for all cases, indicating that the GRMs estimated the parameters accurately.

**Figure 1:**
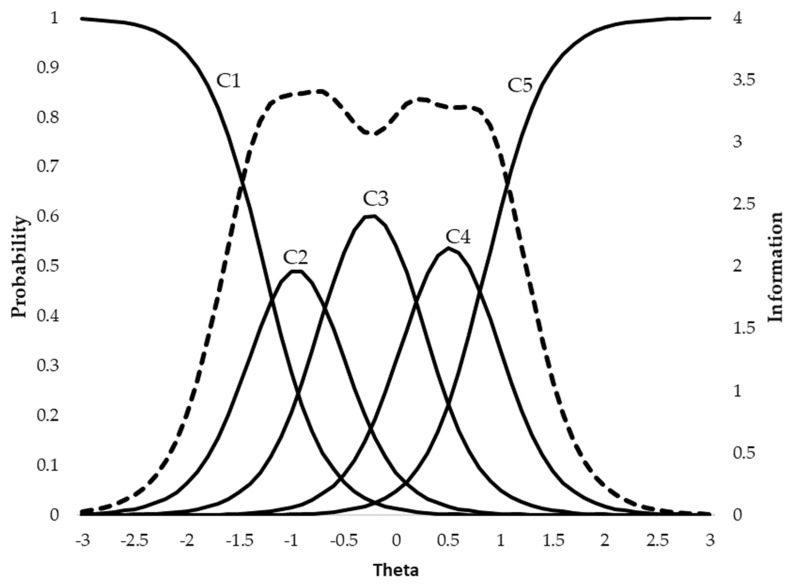
Rating scale categories curves and item information. Solid lines represent the probability of each rating scale category (C1, C2, etc.) conditional to the theta level. The dashed line represents the item information function.

Figure 1 depicts, as an example, the characteristic response curves for Item 7 (“following classroom and school rules”) of the SPA scale. The abscissa axis represents the latent variable theta (M = 0; SD = 1). Five curves are plotted per item (solid lines), each one showing the probability, represented on the left ordinate axis, of belonging to each of the response categories. Thus, in the commented item, for the most probable response to be “full physical assistance” (Category 5), the child/adolescent must present a level of support needs greater than 0.86 standard deviations above the mean. In contrast, the most probable response category for a child situated in the mean (Theta = 0) would be “verbal/gestural prompting” (Category 3). The dashed line represents the zone of the latent variable in which the item is most informative (i.e., it can discriminate between participants with different levels of extraordinary support needs). For this item, the peak of information is approximately between −1.5 and +1 theta scores, thus covering a range of 2.5 standard deviations around the mean of the latent variable.

**Figure 2:**
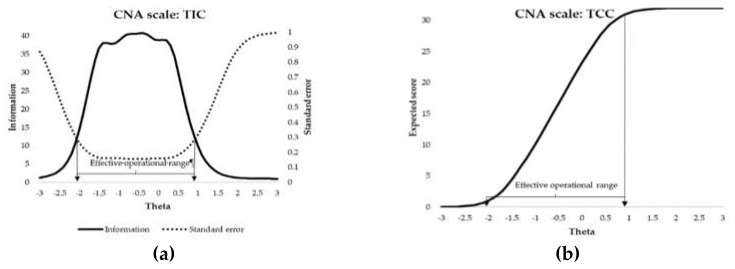
CNA scale: **(a)** test information curve and standard error; **(b)** Test characteristic curve. CNA = Community and neighborhood activities.

Figure 2 shows the test information curve (TIC; panel a) and the test characteristic curve (TCC; panel b) of the CNA scale. The TIC is the result of adding the information curves of all the items of the scale, and it represents the amount of information provided by the test, conditional to the level in the latent variable (theta, depicted in the abscissa axis). The figure also includes the standard error of measurement (dashed line), which represents the reliability of the test along the theta continuum (right ordinate axis). In the CNA scale, the maximum information and reliability were observed at the peak of the TIC approximately between −1.5 and 0.25 theta scores. The effective operational range (EOR) is the area of the variable that is productive for the measure, which on this scale is between −2.1 and +0.9 theta scores. Consequently, the scale was not reliable to assess children with very low support needs (below −2.1 standard deviations) or children with moderate-high or high support needs (above +0.9 standard deviations). Panel b in Figure 2 shows the TCC, which represents the relationship predicted by the model between the raw scores in the CNA scale (ordinate axis) and the latent level of support needs (theta in the abscissa axis). The EOR can be observed clearly in this panel—above the theta score of +0.9 the scale has a strong ceiling effect, where all children obtained the same score independently of their level in the latent trait of support needs; therefore, the scale was not capable of discerning between children with intense extraordinary support needs. Results concerning the TIC, the TCC, and the EOR (as well as the ceiling effect) were similar for all the scales (data and figures for the TIC, the TCC, and the EOR of the HLA, SPA, SLA, HSA, SA, and AA scales are available upon request to the contact author).

An interesting question to assess the utility of scales is how many of the assessed children obtained scores that fell out of the EOR (in other words, for which proportion of the participants the scale has been reliable and useful). Table 5 shows, for each scale, the EOR, as well as the number of children and adolescents with ID who fell out of the EOR and, consequently, were assessed with questionable reliability. The number of participants with scores below the EOR was low (between 0.3% and 4.5%, M = 1.3%). However, the proportion of participants located in the upper EOR was substantially higher (between 13% and 31%, M = 19%). It was explored whether the probability that a child was observed in the upper EOR had any relationship with grouping variables such as gender, age, and degree of disability. The degree of disability was the only variable that showed a strong and consistent relationship. Children with severe/profound ID had more probability (*p* > 0.01) of obtaining scores above the upper EOR for all the scales, with very strong size effects, between Phi = 0.44 (odds ratio = 19.4) in the SPA scale and Phi = 0.58 (odds ratio = 28.9) in the CNA scale. Table 5 also shows the proportion of children and adolescents with severe/profound ID who were observed above the upper EOR (between 74% for SLA and 93% for SA and AA).

## 4. Discussion

Using a large sample of children and adolescents with ID, the present authors applied the GRM method [38] to estimate the discrimination, location, and information functions of the 61 items of the SIS-C [17]. All the items showed high discrimination and information values, and none showed problems that required their removal or review. Considering the discrimination and location of the items together, the results of the IRT scale scores estimations showed substantial variability between individuals, with a score of approximately -2.5 to +1 standard deviations around the latent mean. This result suggests that the SIS-C scales have very good accuracy at low and medium ranges of support needs, but that they may be unreliable at high levels. Prior to drawing a discussion on the findings of this work, it is necessary to acknowledge that the training and expertise of the interviewers selected by each organization, as well as the possible relationship between the interviewer and the assessed child/adolescent with ID, may have influenced the results of this study. Notwithstanding this, although any possible disparity between the interview style followed by the interviewers selected by the organizations versus that followed by the research team may have supposed a bias source, the present authors designed and implemented a training program to make the interviews as homogeneous as possible, always following the SIS-C guidelines for the interview process [17].

The proportion of children and adolescents with extraordinary support needs located in the low reliability zone was between 13% (HLA) and 31% (SLA). To better illustrate this result, we can take as an example the SLA scale—the scores discriminated very well between participants with support needs between -−2.5 and +0.5 standard deviations along the latent continuum, but remained constant in the range from +0.5 to +3 standard deviations, giving rise to a strong and extensive ceiling effect (i.e., two children with substantially different support needs would obtain the same observed scores). This ceiling effect was observed for the seven scales. It does not seem to be related to the age or gender of the participants, supporting the decision to use the same set of items for all age bands, as suggested in previous research [44,45,46]. The probability of being observed in the low discrimination range strongly depended on the level of ID given that, on average, 87% of the cases located in the ceiling effect area had severe/profound ID.

One central question is to what extent a ceiling effect poses a problem for the usefulness of the SIS-C [17]. The presence of wide ranges of low discrimination can only be regarded as a deficiency in a tool if it interferes with the goals for which it was developed. In clinical diagnosis scales, it is common to observe strong floor effects because the indicators must concentrate their discriminative power at high levels of the variable, which is useful for classifying persons into groups (e.g., affected and unaffected). For example, in an adaptive behavior scale whose goal is the diagnosis of ID, it is possibly not applicable to evaluate in detail medium and low deficit levels, so the items will be designed to provide maximum information in high areas of the variable—those relevant for diagnosis. However, the aim of the SIS-C is not to diagnose but to assess the pattern and intensity of needs that serve for supports planning [8,17]. Consequently, the SIS-C is expected to accurately measure a wide range of intensity of support needs so that most people are assessed accurately enough regardless of the intensity of their support needs. Under these circumstances, a strong ceiling effect may interfere with the goals of the SIS-C, which implies potentially important consequences for both research and practice.

Regarding research, the low variability in a significant proportion of the participants may bias the results of the data analyses. The support needs in the ceiling area will tend to behave as a constant rather than as a variable, inhibiting the possibility of analyzing heterogeneity in an important part of the sample and its relationship with other predictor, criterion, or grouping variables. This may bias research results in persons with high levels of support needs. An example is research that focuses on investigating the impact of support needs on QoL by using a linear or nonlinear regression model. For this, it is necessary that the criterion variable has variance—if, for a large proportion of participants the criterion behaves like a constant, the regression weights will be unable to capture part of the variability of the criterion, altering the results and making their interpretation difficult. Another example is studies analyzing the impact of supports planning—possibly, any real impact of the program would be hidden because of the problem of measurement discrimination in high areas of the variable.

As regards the applied field, support needs assessment obeys the need to understand the pattern and intensity of the extraordinary support that children and adolescents with ID require with the aim of providing personalized supports that enhance their functioning and QoL [1], such as personalized education plans that include goals beyond literacy [22] and the use of personalized support plans to improve personal outcomes in inclusive settings [23]. However, for children and adolescents with ID located in the ceiling effect area, no pattern of support needs will be obtained—rather, homogeneous scores where all or almost all the indicators acquire the maximum score. For these children, the SIS-C is not able to capture individual differences, does not allow recognition of the real pattern of support needs, and ultimately does not provide useful information for planning personalized programs or for evaluating the effectiveness of the intervention. These issues hinder efficiency in supports planning regarding children and adolescents with ID, which, in turn, may have negative effects on the efforts made to enhance their inclusion and QoL. The problem is that the main environmental demands that interact with their capacities are related to educational contexts and activities. It is precisely these contexts on which economic cuts have had the greatest impact over the last decade, which makes it especially necessary to have instruments that allow different levels of support needs to be adequately discerned to support more efficient decision-making in resources and supports implementation to enhance QoL [8].

There are solutions to the research and applied problems described above. In instruments aimed at measuring a wide spectrum of individual differences (such as the SIS-C), it is common to observe poor discrimination in extreme areas of the latent variable, as usually happens, for example, in the assessment of general intelligence [47]. In practice, this is not relevant if the tails of the distribution are short, contain few people, and the scale is capable of accurately capturing individual differences in most of those assessed. That said, the SIS-C assesses low to medium levels of support needs with good precision, but it presents significant discrimination difficulties at high levels. To solve this problem, it is necessary to change the traditional idea of fixed-length scales by the idea of personalized instruments based on pools of items calibrated using IRT models. As has been shown in this study, it is possible to know how well each item differentiates between people who are at different levels of the latent variable, so it is possible to combine different items to maximize information in different ranges of the assessed continuum. Therefore, it would be possible to develop a pool of indicators with high discrimination and with location parameters distributed over a range of the latent variable as wide as necessary. In the IRT, the true position of the assessed person in the latent variable does not depend on the specific set of items administered [31]; therefore, this calibrated pool of items would allow the development of instruments adapted to the assessment needs of each person or group (e.g., by applying only discriminatory items in the moderate-high range of support needs for persons with severe/profound ID). This would make it possible to minimize the problem of the ceiling effect, improve substantially the assessment accuracy for all the ranges of support needs (which would facilitate more efficient supports planning), and reduce the length of the test without prejudice to reliability, as well as further the development of high-quality and efficient assessment systems such as computerized adaptive tests [48]. For the case of children with severe/profound ID, developing and calibrating such set of indicators would translate into better opportunities to obtain positive outcomes regarding the different environments considered by the SIS-C. These indicators would allow to obtain personalized profiles of support needs in which to base a thoughtful provision of supports aligned with their vital expectations, something that, in turn, can help to overcome the idea of the “more is better” in supports planning and resources allocation, an idea which is especially anchored regarding persons with severe/profound ID and which has been used in (not a few) occasions to neglect the provision of opportunities with those with extensive support needs [49].

## 5. Conclusions

The SIS-C items showed high discrimination and information capacity; therefore, its modification does not appear necessary. However, all scales showed discrimination problems in children and adolescents with high support needs, especially people with severe/profound ID. This ceiling effect may be an important obstacle for both research and practice involving the SIS-C. A key objective for future research to improve the SIS-C is to design reliable and discriminatory indicators in areas of high intensity of support needs.

## Figures and Tables

**Table 1:** Supports Intensity Scale–Children’s Version (SIS-C) domains’ descriptions and measurement methods for the ‘Support Needs Scale’ section.

**SIS-C Activity Domains**		
HLA includes nine household activities. Examples include “eating” and “using electronic devices.”		
CNA is composed of eight activities involved in being a member of the neighborhood or the community. Exemple activities include “using public services” and “shopping.”		
SPA incorporates nine activities linked to school participation. “Following classroom and school rules” and “participating in activities in common school areas” are examples of activities in this domain.		
SLA is made up of nine activities associated with acquiring knowledge and/or skills while attending school (e.g., “learning” and “completing homework assignments”).		
HSA involves eight activities linked to assuring safety and health across home, school, and community. Examples are “maintaining physical fitness” and “responding in emergency situations.”		
SA includes nine activities related to social interactions with others. Among these activities, examples are “making and keeping friends” and “maintaining conversation.”		
AA are core activities relevant to being the causal agent over one’s life. Exemple activities involve “making personal choices and decisions.”		
**Measurement Methods’ Rating Scales for Each SIS-C Activity**		
**Type of Support**	**Frequency of Support**	**Daily Support Time**
0 = None	0 = Negligible	0 = None
1 = Monitoring	1 = Infrequently	1 = Less than 30 minutes
2 = Verbal/gestural prompting	2 = Frequently	2 = 30 minutes to less than 2 hours
3 = Partial physical assistance	3 = Very frequently	3 = 2 hours to less than 4 hours
4 = Full physical assistance	4 = Always	4 = 4 hours or more

Note. HLA = Home living activities; CNA = Community and neighborhood activities; SPA = School participation activities; SLA = School learning activities; HSA = Health and safety activities; SA = Social activities; AA = Advocacy activities.

**Table 2:** Sociodemographic and clinical variables of the assessed children and adolescents.

**Sociodemographic Variables**	**N**	**%**	**Clinical Variables**	**N**	**%**
**Gender**			**Etiology of ID**		
Male	528	64.86	Non-specific	317	38.94
Female	286	35.14	Down’s Syndrome	111	13.64
**Total**	814	100	Autism Spectrum Disorder	248	30.47
**^1^ Autonomous Community**			Cerebral Palsy	101	12.42
Andalusia	113	13.88	Rare diseases	35	4.29
Aragon	23	2.83	Co-occurrence	2	0.24
Canary Islands	86	10.56	Total	814	100
Cantabria	27	3.32	**^2^ Adaptive behavior limitations**		
Castile-La Mancha	101	12.41	Mild	174	21.38
Castile and Leon	154	18.92	Moderate	314	38.57
Extremadura	23	2.83	Severe	200	24.57
Galicia	50	6.14	Profound	67	8.23
Madrid	145	17.81	N/A	59	7.25
Murcia	28	3.44	Total	814	100
Valencia	64	7.86	**^2^ Intellectual functioning limitations**		
Total	814	100	Mild	206	25.31
**Primary language**			Moderate	290	35.63
Spanish	784	96.32	Severe	195	23.96
Galician	2	0.24	Profound	65	7.98
Romanian	2	0.24	N / A	58	7.12
Arabian	3	0.36	Total	814	100
Valencian	8	0.98	**Assistive technology use**		
English	1	0.13	Yes	157	19.29
Ukrainian	1	0.13	No	657	80.71
SSL	1	0.13	Total	814	100
Missing	12	1.47	**Sensory condition associated**		
Total	814	100	Yes	91	11.18
**Age cohort**			No	723	88.82
5–6	110	13.51	Total	814	100
7–8	108	13.27	**Physical condition associated**		
9–10	100	12.28	Yes	174	21.38
11–12	148	18.18	No	640	78.62
13–14	195	23.95	Total	814	100
15–16	153	18.81	**Language condition associated**		
Total	814	100	Yes	389	47.79
**Schooling**			No	425	52.21
Special education	493	60.56	Total	814	100
General education	179	21.99			
Combined	129	15.84			
Missing data	13	6.61			
Total	814	100			
**Educational stage**					
Elementary	110	13.51			
Primary	356	43.73			
Secondary	348	42.76			
Total	814	100			
**Living**					
Family home	777	95.45			
Foster family home	9	1.11			
Small group home (< 7)	7	0.86			
Midsize group home (7–15)	9	1.11			
Large residential facility (> 15)	3	0.36			
Missing data	9	1.11			
Total	814	100			

Note. SSL = Spanish sign language. ^1^ Autonomous Community = Region with administrative and legislative autonomy of the Kingdom of Spain. ^2^ Adaptive behavior and intellectual functioning limitations are based on educational records, not actual scores on standardized assessments. N / A = Not available.

**Table 3:** Results of dimensionality, reliability, and local independence analyses.

Scale	EV-FA	PA	ECV	MIREAL	FDI	H-G	MR
HLA	0.87	1	0.95	0.17	0.98	0.97	0.97
CAN	0.83	1	0.96	0.14	0.98	0.97	0.97
SPA	0.79	1	0.94	0.15	0.98	0.97	0.97
SLA	0.84	1	0.95	0.17	0.99	0.98	0.98
HAS	0.81	1	0.95	0.19	0.98	0.96	0.97
SA	0.79	1	0.94	0.18	0.98	0.97	0.97
AA	0.84	1	0.95	0.18	0.99	0.98	0.98

Note. HLA = Home living activities; CNA = Community and neighborhood activities; SPA = School participation activities; SLA = School learning activities; HSA = Health and safety activities; SA = Social activities; AA = Advocacy activities; EV-FA = Variance explained by the first eigenvalue; PA = Number of factors recommended by parallel analysis; ECV = Explained common variance; MIREAL = Mean of item absolute residuals; FDI = Factor determinacy index; H-G = Latent index of replicability; MR = Marginal reliability of estimated a posteriori scores.

**Table 4:** Model parameters.

Scale	Item	α	α s.e.	β1	β1 s.e.	β2	β2 s.e.	β3	β3 s.e.	β4	β4 s.e.
HLA	1	3.41	0.20	−1.66	0.08	−0.97	0.05	−0.14	0.04	0.61	0.05
	2	3.79	0.22	−0.79	0.05	−0.21	0.04	0.22	0.04	0.98	0.06
	3	5.84	0.39	−1.21	0.06	−0.64	0.04	−0.26	0.04	0.45	0.04
	4	5.05	0.32	−1.04	0.05	−0.52	0.04	−0.19	0.04	0.66	0.05
	5	4.88	0.30	−0.68	0.04	−0.17	0.04	0.12	0.04	0.74	0.05
	6	2.21	0.13	−0.31	0.05	0.30	0.05	0.89	0.06	1.53	0.08
	7	2.98	0.17	−0.97	0.06	−0.35	0.05	0.21	0.05	0.69	0.05
	8	2.42	0.14	−1.17	0.07	−0.45	0.05	0.20	0.05	0.98	0.07
	9	2.55	0.15	−1.21	0.07	−0.47	0.05	−0.09	0.05	0.58	0.06
CNA	1	4.11	0.25	−1.45	0.07	−0.76	0.05	−0.37	0.04	0.24	0.05
	2	3.70	0.21	−1.35	0.06	−0.75	0.05	−0.18	0.04	0.47	0.05
	3	3.62	0.21	−1.34	0.06	−0.66	0.05	0.05	0.05	0.75	0.06
	4	4.91	0.31	−1.59	0.07	−0.99	0.05	−0.45	0.04	0.13	0.05
	5	5.04	0.32	−1.37	0.06	−0.81	0.05	−0.30	0.04	0.28	0.05
	6	3.90	0.23	−1.76	0.08	−1.07	0.05	−0.42	0.04	0.22	0.05
	7	3.42	0.20	−1.47	0.07	−0.85	0.05	−0.14	0.05	0.39	0.05
	8	4.71	0.29	−1.49	0.06	−0.75	0.05	−0.24	0.04	0.39	0.05
SPA	1	3.00	0.18	−1.75	0.09	−1.19	0.06	−0.47	0.05	0.20	0.05
	2	4.63	0.28	−1.24	0.06	−0.60	0.04	0.02	0.04	0.67	0.05
	3	4.37	0.27	−1.46	0.06	−0.83	0.05	−0.23	0.04	0.45	0.05
	4	2.67	0.16	−1.48	0.08	−0.75	0.05	−0.33	0.05	0.27	0.05
	5	4.57	0.27	−0.97	0.05	−0.40	0.04	0.20	0.05	0.83	0.06
	6	2.43	0.16	−2.18	0.12	−1.40	0.07	−0.52	0.05	−0.09	0.05
	7	3.45	0.19	−1.26	0.06	−0.64	0.05	0.17	0.05	0.86	0.06
	8	3.92	0.23	−1.02	0.05	−0.51	0.04	0.09	0.05	0.60	0.05
	9	4.22	0.25	−1.06	0.05	−0.61	0.04	0.09	0.04	0.58	0.05
SLA	1	4.55	0.33	−2.04	0.13	−1.50	0.09	−0.69	0.06	−0.15	0.05
	2	5.49	0.41	−2.06	0.13	−1.51	0.09	−0.64	0.05	0.03	0.05
	3	5.28	0.41	−2.03	0.13	−1.56	0.10	−0.79	0.06	−0.26	0.05
	4	5.57	0.42	−1.88	0.11	−1.40	0.09	−0.61	0.05	0.02	0.05
	5	3.56	0.24	−1.81	0.11	−1.23	0.08	−0.50	0.05	0.37	0.05
	6	4.26	0.30	−2.02	0.13	−1.54	0.10	−0.59	0.05	0.02	0.05
	7	5.25	0.40	−1.85	0.11	−1.46	0.09	−0.67	0.05	−0.23	0.05
	8	3.76	0.26	−1.81	0.11	−1.32	0.08	−0.62	0.06	0.06	0.05
	9	3.71	0.26	−1.90	0.12	−1.23	0.08	−0.53	0.05	0.07	0.05
HSA	1	3.07	0.18	−1.11	0.06	−0.67	0.05	0.04	0.05	0.59	0.06
	2	3.36	0.20	−1.39	0.07	−1.01	0.06	−0.29	0.05	0.26	0.05
	3	3.20	0.19	−1.63	0.08	−1.09	0.06	−0.13	0.05	0.49	0.06
	4	4.53	0.29	−1.50	0.07	−0.94	0.05	−0.25	0.05	0.29	0.05
	5	4.34	0.31	−1.66	0.08	−1.21	0.06	−0.75	0.05	−0.30	0.05
	6	4.82	0.36	−1.75	0.08	−1.30	0.06	−0.78	0.05	−0.35	0.05
	7	4.04	0.27	−1.72	0.08	−1.19	0.06	−0.62	0.05	−0.19	0.05
	8	3.70	0.23	−1.52	0.07	−0.98	0.06	−0.39	0.05	0.15	0.05
SA	1	4.08	0.24	−1.32	0.06	−0.78	0.05	0.06	0.05	0.81	0.06
	2	2.90	0.17	−1.24	0.06	−0.68	0.05	0.03	0.05	0.64	0.06
	3	4.35	0.27	−1.34	0.06	−0.88	0.05	−0.11	0.04	0.31	0.05
	4	4.19	0.27	−1.75	0.08	−1.18	0.05	−0.34	0.04	0.03	0.05
	5	2.63	0.15	−1.42	0.07	−0.87	0.06	0.06	0.05	0.81	0.06
	6	4.67	0.29	−1.35	0.06	−0.88	0.05	−0.19	0.04	0.23	0.05
	7	4.48	0.27	−1.39	0.06	−0.88	0.05	−0.13	0.04	0.44	0.05
	8	2.70	0.15	−1.13	0.06	−0.49	0.05	0.09	0.05	0.66	0.06
	9	3.09	0.20	−1.67	0.08	−1.13	0.06	−0.51	0.05	−0.15	0.05
AA	1	2.85	0.17	−1.05	0.07	−0.46	0.05	0.43	0.06	1.12	0.07
	2	5.11	0.35	−1.72	0.09	−1.24	0.06	−0.48	0.05	−0.09	0.05
	3	5.96	0.43	−1.81	0.09	−1.36	0.07	−0.49	0.05	0.02	0.05
	4	3.61	0.22	−1.66	0.09	−1.11	0.06	−0.07	0.05	0.48	0.06
	5	4.13	0.27	−1.45	0.07	−0.96	0.06	−0.34	0.05	0.10	0.05
	6	5.48	0.38	−1.68	0.08	−1.25	0.06	−0.51	0.05	−0.07	0.05
	7	3.26	0.19	−1.21	0.07	−0.74	0.06	0.09	0.05	0.74	0.06
	8	4.59	0.31	−1.70	0.09	−1.26	0.07	−0.45	0.05	−0.09	0.05
	9	5.06	0.34	−1.89	0.10	−1.40	0.07	−0.57	0.05	−0.02	0.05

Note. HLA = Home living activities; CNA = Community and neighborhood activities; SPA = School participation activities; SLA = School learning activities; HSA = Health and safety activities; SA = Social activities; AA = Advocacy activities; s.e. = Standard error of estimation.

**Table 5:** Effective operational ranges and scores distribution.

	HLA	CNA	SPA	SLA	HSA	SA	AA
EOR lower bound	−1.4	−2.1	−1.9	−2.5	−2.2	−2	−2.2
EOR upper bound	1.3	0.9	1.2	0.5	0.8	1.1	0.7
EOR range	2.7	3	3.1	3	3	3.1	2.9
% children below EOR	4.5	0.6	1.2	0.3	0.6	1.2	0.8
% children above EOR	13	21	13	31	22	14	21
% children with S / P ID above upper EOR	90	88	87	74	83	93	93

Note. HLA = Home living activities; CNA = Community and neighborhood activities; SPA = School participation activities; SLA = School learning activities; HSA = Health and safety activities; SA = Social activities; AA = Advocacy activities; S / P ID = Severe / Profound Intellectual disability.
